# Some Paralytic Deformities in Children

**Published:** 1907-04-27

**Authors:** 


### SOME PARALYTIC DEFORMITIES IN CHILDREN.

### TRANSPLANTATION OF TENDONS. FIXATION OF JOINTS BY EXCISION.

The management of deformities due to anterior poliomyelitis has undergone interesting changes during recent years. The transplantation of tendons has proved so successful as to revolutionise this branch of pediatrics. Why should a partially paralysed limb be condemned to carry cumbrous instruments if the functions of the joints can be restored by other means? This can be brought about by a carefully-planned transplantation of tendons of the remaining set of healthy muscles into the tendons of the paralysed group. If this is done, instrumentation, which further impedes the circulation in an already cold, blue, and weak limb, may in a great many cases be dispensed with. Not only is this desirable on account of the weight of the instrument, but on account also of its liability to get out of order, constantly requiring readjustment and renovation, and thereby being the cause of considerable expense. The most promising cases for tendon anastomosis are those in which the paralysis is limited to one muscle group; either the extensors of the toes, or the peronei, or the flexors, or the muscles acting on the tendo Achillis. After the paralysis has existed over a year or two, deformities arise from the unopposed action of the healthy muscles; and the healthy tendons thus becoming abnormally short, require division before the joint can be replaced in its normal position. If tenotomy alone is performed, and the limb put up in plaster or other fixative, the deformity will eventually recur unless the correction is maintained by mechanical control.

Instead, therefore, of merely performing tenotomy, a tendon of the unparalysed group is selected and implanted so that its muscle can act upon the tendon of the paralysed muscle; not only is the deformity overcome, but its recurrence is prevented without resort to instrumentation.

To take an instance. A boy of five years was brought by his mother on account of flexion of the great toe, which caught in the ground in walking when bare-footed. On examination this was found to be due to inability actively to extend the toe in the very slightest degree. All the other toes could be extended, and no other muscle group was completely paralysed. The tendon of the extensor longus hallucis was implanted into the innermost tendon of the unparalysed extensor longus digitorum. The result was complete control of the great toe, so that it no longer caught on the ground in walking.

There is a wide range in the extent of the paralysis between that of a single muscle, as in the case just cited, and complete paralysis of the limb. Even in many of the worst cases, however, something may be done to mitigate the condition.

In severe cases the flail-like limb at first sight appears to be completely paralysed. Careful examination by inspection, even without the aid of electrical reactions of the nerves and muscles, may reveal one set of muscles, perhaps the glutei or the adductors to be unaffected, and the hamstrings only partially affected, while the extensors of the knee and other muscles of the leg are completely paralysed. In such a case the biceps tendon of the hamstring group and the adductor magnus tendon may be isolated, divided low down, and brought to the front of the knee, where they are sutured to the extensor tendon of the joint.

When the muscles are completely paralysed the joints may be fixed by excision of the articular cartilage so as to bring about osseous union. This has been done in the knee and sub-astragaloid joints, leaving movement to take place in the ankle joint, upon which a dorsiflexion spring may be made to act.

These cases require very careful investigation before anything can be done, and the after-treatment must be particularly painstaking.

